# Association of daily composition of physical activity and sedentary behaviour with incidence of cardiovascular disease in older adults

**DOI:** 10.1186/s12966-021-01157-0

**Published:** 2021-07-12

**Authors:** Manasa S. Yerramalla, Duncan E. McGregor, Vincent T. van Hees, Aurore Fayosse, Aline Dugravot, Adam G. Tabak, Mathilde Chen, Sebastien F. M. Chastin, Séverine Sabia

**Affiliations:** 1 Université de Paris, Inserm U1153, Epidemiology of Ageing and Neurodegenerative diseases, 10 Avenue de Verdun, 75010 Paris, France; 2 School of Health and Life Sciences, Glasgow Caledonian University, Glasgow, Scotland, UK; 3 Biomathematics and Statistics Scotland, Edinburgh, UK; 4 Accelting, Almere, Netherlands; 5 Department of Epidemiology and Public Health, University College London, London, UK; 6 Department of Internal Medicine and Oncology, Semmelweis University, Faculty of Medicine, Budapest, Hungary; 7 Department of Public Health, Semmelweis University, Faculty of Medicine, Budapest, Hungary; 8 Department of Movement and Sports Sciences, Ghent University, Ghent, Belgium

**Keywords:** Cardiovascular disease, Compositional data analysis, Light intensity physical activity, Longitudinal cohort, Moderate-to-vigorous physical activity, Older adults, Sedentary behaviour

## Abstract

**Background:**

Moderate-to-vigorous physical activity (MVPA) is proposed as key for cardiovascular diseases (CVD) prevention. At older ages, the role of sedentary behaviour (SB) and light intensity physical activity (LIPA) remains unclear. Evidence so far is based on studies examining movement behaviours as independent entities ignoring their co-dependency. This study examines the association between daily composition of objectively-assessed movement behaviours (MVPA, LIPA, SB) and incident CVD in older adults.

**Methods:**

Whitehall II accelerometer sub-study participants free of CVD at baseline (*N* = 3319, 26.7% women, mean age = 68.9 years in 2012–2013) wore a wrist-accelerometer from which times in SB, LIPA, and MVPA during waking period were extracted over 7 days. Compositional Cox regression was used to estimate the hazard ratio (HR) for incident CVD for daily compositions of movement behaviours characterized by 10 (20 or 30) minutes greater duration in one movement behaviour accompanied by decrease in another behaviour, while keeping the third behaviour constant, compared to reference composition. Analyses were adjusted for sociodemographic, lifestyle, cardiometabolic risk factors and multimorbidity index.

**Results:**

Of the 3319 participants, 299 had an incident CVD over a mean (SD) follow-up of 6.2 (1.3) years. Compared to daily movement behaviour composition with MVPA at recommended 21 min per day (150 min/week), composition with additional 10 min of MVPA and 10 min less SB was associated with smaller risk reduction – 8% (HR, 0.92; 95% CI, 0.87–0.99) – than the 14% increase in risk associated with a composition of similarly reduced time in MVPA and more time in SB (HR, 1.14; 95% CI, 1.02–1.27). For a given MVPA duration, the CVD risk did not differ as a function of LIPA and SB durations.

**Conclusions:**

Among older adults, an increase in MVPA duration at the expense of time in either SB or LIPA was found associated with lower incidence of CVD. This study lends support to public health guidelines encouraging increase in MVPA or at least maintain MVPA at current duration.

**Supplementary Information:**

The online version contains supplementary material available at 10.1186/s12966-021-01157-0.

## Background

Physical activity is a modifiable risk factor for cardiovascular diseases (CVD) [1, 2], with 17 to 25% lower CVD risk among those following the current recommended 150 min per week of moderate-to-vigorous physical activity (MVPA) [3]. These recommendations are not met by the majority of the population, particularly older adults [4] in part due to declining physiological ability to perform higher intensity activity [5]. Older adults spend around two-thirds of the day in sedentary behaviour (SB) [6] which is increasingly thought to be an independent risk factor for CVD [7–9]. Light intensity physical activity (LIPA) such as strolling may be easier than MVPA [10] and could potentially confer some benefits in those not fit enough to engage in physical activity at higher intensity [11].

The evidence on the association between multiple movement behaviours during the day (i.e. SB, LIPA, MVPA) and CVD is primarily based on self-reported physical activity [12], which are subject to recall and response bias [13]. Further limitations of such data include their inability to capture incidental, short periods of movements and light intensity activities that are spread over the day and thus less easy to report with accuracy [12–14]. Such measurements are likely to affect precision of estimates of the associations between movement behaviours and health outcomes [15]. Prospective studies are beginning to use movement sensor devices such as accelerometers to objectively measure duration in movement behaviours and assess their associations with incident CVD [11, 16–20]. These studies have found higher duration of MVPA to be associated with reduced risk of CVD [16–18, 20], while results on the impact of LIPA [11, 16–18, 20] and SB [16–20] are inconsistent.

Each day is limited in time, with increase in time spent in one movement behaviour done at the expense of time in other movement behaviours [21]. Most prospective studies using objective measures have not accounted for this co-dependency and treated SB [19, 20], LIPA [11, 20, 22] and MVPA [3, 20] as independent behaviours. Recent epidemiological research calls to account for the relative nature of movement behaviours and to use appropriate statistical methods to deal with such data [23, 24]. We aim to use an innovative approach, the compositional Cox regression [25] to better examine how different compositions of SB, LIPA, and MVPA in a waking day are associated with incident CVD in older adults. This approach explicitly considers the compositional nature of movement data, that is the durations in SB, LIPA, and MVPA are part of a composite whole corresponding to the waking period of a day.

## Methods

### Study participants

The Whitehall II study is an ongoing prospective cohort established in 1985–1988 among 10,308 London-based civil servants (67% males) aged 35–55 years [26]. Since the inception of the study, sociodemographic, lifestyle and health-related factors have been assessed using questionnaires and clinical examinations. Follow-up assessments have taken place approximately every 4–5 years, with the latest wave completed in 2015–2016. Participants provided written informed consent. Research ethics approval was obtained from the University College London ethics committee (reference number 85/0938), renewed at each contact. The accelerometer sub-study was undertaken during the 2012–2013 wave of data collection for participants seen at the London clinic and for those living in the South-Eastern regions of England who underwent clinical examination at home.

### Measures

#### Movement behaviours

Participants without contraindications (i.e., allergies to plastic or metal, travelling abroad in the following week) were asked to wear a tri-axial accelerometer (GENEActiv Original; Activinsights Ltd., Kimbolton, UK) on their non-dominant wrist during 9 consecutive days over 24 h. Data was sampled at 85.7 Hz, with acceleration expressed relative to gravity (1 *g* = 9.81 m/second^2^).

Accelerometer data was processed in R software by using GGIR package [27] version 2.0–1 (https://github.com/wadpac/GGIR/releases/tag/v2.0-1). Data were corrected for calibration error [28] and Euclidean Norm of raw accelerations Minus 1 (ENMO) with negative numbers rounded to zero were calculated [29]. This metric has been shown to be a valid measure of time spent in metabolic equivalent of task (MET) levels as measured by indirect calorimetry [30]. Sleep periods were then detected using a validated algorithm guided by sleep log [31]. Data from the first waking up (day 2) to waking up on the day before the last day (day 8) were used, corresponding to 7 full days. Waking period was defined as the period between waking and onset of sleep. Participants were included in the analysis if they had daily wear time ≥ 2/3 of waking hours, for at least 2 weekdays and 2 weekend days [32]. Non-wear period among valid days was corrected based on a previously reported algorithm [29]. As there is no gold standard to classify movement behaviours in older adults, we referred to cut-points from a study where adult participants undertook series of activities in a laboratory and mimic free-living posture/behaviours eliciting average accelerations similar to that observed in free living situations [33]. These cut-points are in agreement with a more recent study among older adults that showed good classification accuracy based on oxygen consumption during nine laboratory-based activities of daily living [34]. Based on these studies, movement behaviour during waking period was classified as SB when average acceleration over a 60-s epoch was < 40 milligravity (m*g*), 40–99 m*g* for LIPA and ≥ 100 m*g* for MVPA [33, 34]. The daily time in each movement behaviour was calculated as the mean of measures over 7 days. For those with < 7 valid days, a weighted average was computed using data on weekend and week days [32]. Reliability of acceleration measures was assessed among 66 participants using retest data assessed on average for 26.5 (SD = 4.6) days after the first measure. There was a good test-retest reliability for all movement behaviours with strong correlation between the two measures (Pearson’s r = 0.81 for SB, 0.77 for LIPA and 0.75 for MVPA).

#### Incidence of CVD

Incident CVD was defined as the first occurrence of fatal or nonfatal coronary heart disease (CHD), stroke or heart failure. Nonfatal events were traced from the Hospital Episode Statistics (HES) database using each participant’s unique National Health Services (NHS) identification number based on the International Classification of Diseases (ICD) codes for CHD (ICD-10 codes I20–25), stroke (ICD-10 codes I60–I64) and heart failure (ICD-10 code I50). CHD and stroke cases were also determined using Whitehall II study-specific 12-lead resting electrocardiogram recording and MONICA-Augsburg stroke questionnaire, respectively [26]. Further details of validation of CVD cases are provided in a separate publication [35]. CVD-related deaths were drawn from the UK national mortality register (NHS Central Registry).

#### Covariates

Covariates were assessed by questionnaire or at clinical examination during the 2012–2013 wave. Sociodemographic variables included age, sex, ethnicity (white, non-white), marital status (married/cohabitating, divorced/widowed/single), education (≤primary school, lower secondary school, higher secondary school, university, higher degree) and last occupational position (high, intermediate, low). Lifestyle factors included alcohol consumption (0, 1–14, > 14 units per week), smoking status (current and recent ex-(less than 5 years) smokers, long-term ex-smokers, never smokers), and fruits & vegetables intake (less than once daily, once daily, more than once daily). Cardiometabolic risk factors included body mass index (BMI; categorized as < 24.9, 25–29.9, and ≥ 30 kg/m^2^), hypertension (systolic/diastolic blood pressure ≥ 140/90 mmHg or use of antihypertensive drugs), prevalent diabetes (fasting glucose ≥7.0 mmol/l or self-reported doctor diagnosed diabetes or use of diabetes medication or hospitalizations ascertained through record linkage to the HES (ICD-9 codes 250 or ICD-10 code E11)), hyperlipidaemia (low-density lipoproteins (LDL) > 4.1 mmol/l or use of lipid-lowering drugs) assessed at the clinical examination, and multimorbidity index (calculated as the count of the following chronic conditions: cancer, arthritis, chronic obstructive pulmonary disease, depression, Parkinson disease, and dementia; assessed using HES records and Whitehall II questionnaires as well as mental health records for depression).

### Statistical analysis

Compositional data analysis was used to account for the co-dependency of movement behaviours during waking period [21]. This method assumes relative distribution of the movement behaviours and reduces the three-parts composition (SB, LIPA, MVPA) noted as z, into two exposure variables by transforming them into two isometric log-ratio (ilr) coordinates, referred to as z_1_ and z_2_. The following vector of ilr-coordinates was first constructed by sequential binary partition to examine the importance of SB [36]: (Eq. 1)

1 $$ {z}^1=\left(\begin{array}{c}{z}_1^1=\sqrt{\frac{2}{3}}\mathit{\ln}\frac{SB}{{\left( LIPA. MVPA\right)}^{1/2}},\\ {}{z}_2^1=\sqrt{\frac{1}{2}}\mathit{\ln}\frac{LIPA}{MVPA}\end{array}\right) $$

The first coordinate, ${z}_1^1$ represents the ratio of time spent in SB relative to the geometric mean of physical activity (LIPA and MVPA). Second coordinate, ${z}_2^1$ represents time spent in LIPA with respect to MVPA. Rotations of these coordinates, *z*^2^ and *z*^3^, were then used to examine the importance of LIPA relative to SB and MVPA, and the importance of MVPA relative to LIPA and SB (Supplemental Methods Section). In order to facilitate interpretation of the results, daily time spent in SB, LIPA and MVPA were normalized to a 16-h waking day corresponding to the mean duration of waking period in our study population. Zero duration in any of the movement behaviours were imputed using log ratio Expectation–Maximization algorithm implemented using lrEM function in R package *zCompositions* [37]. Analyses were conducted using compositional Cox regression with incident CVD as the outcome [25]. The proportional hazards assumption was checked using the Grambsch–Therneau test statistic [38]. Follow-up time for incident CVD event was from the date of clinical examination at the 2012–2013 wave until the date of CVD (fatal or non-fatal), non-CVD related death to account for competing risks or end of follow-up (31st March 2019), whichever came first. Model 1 included waking day composition of z (z_1_, z_2_), sociodemographic and lifestyle variables. Model 2 was additionally adjusted for cardiometabolic risk factors and multimorbidity index. We examined whether age (< 67.8 versus ≥67.8 years (median split)), sex (men versus women), ethnicity (white versus non-white) and BMI (not obese versus obese) modified associations. Effect modification was investigated by adding the interaction terms “effect modifier*z_1_” and “effect modifier*z_2_” to the fully adjusted model.

Results from the Cox compositional model [25] were used to extract the hazard ratios (HR) for CVD incidence in relation to compositions of movement behaviours (MVPA, LIPA, SB) in a waking day period. To do so, we defined a set of reference movement behaviour compositions to which we compared compositions with 10 (20- and 30-) minutes more time spent in one movement behaviour at the expense of time spent in another movement behaviour, keeping time spent in the third behaviour fixed at the reference composition. This approach is also referred to as reallocation of time between different movement behaviours [25]. First to assess the impact of MVPA duration, we defined three separate reference movement behaviour compositions corresponding to individuals with daily MVPA duration set at 10 min, 21 min (corresponding to the MVPA recommendation of 150 min per week) [39], and the recommended 30 min per day [39]. Time in SB and LIPA for these reference compositions were set at 77 and 23%, respectively, of the remaining waking time, based on the mean proportion of these movement behaviours as observed in the study sample.

To examine whether risk of CVD varied by duration in SB or LIPA, reallocation analyses were conducted using two reference daily compositions of movement behaviours with SB set at 9 h and 14 h corresponding to the 5th and 95th percentile, respectively and MVPA duration set at 10 min. LIPA was set at the remaining waking day by subtracting time spent in SB and MVPA from 16 h. We then examined whether similar findings were found when repeating these analyses with 21 min instead of 10 min of MVPA in the reference composition. All analyses were undertaken using STATA statistical software version 15 (StataCorp, College Station, Texas) and R version 3.6.3 with a two-sided *P* < 0.05 considered statistically significant.

### Sensitivity analyses

The robustness of results was tested in further analyses. One, to examine the potential risk of reverse causation we repeated the primary analysis by excluding incident CVD events occurring within the first 2 years of follow-up. Two, the outcome was defined as incident non-fatal CVD events, where fatal CVD events were censored at date of death but not considered as an event of interest. Three, we repeated analysis by using an alternative cut-off to differentiate SB (< 45 m*g*) from LIPA (45–99 m*g*) [40]. Four, we also provide results using time in SB, LIPA and MVPA without normalizing to 16-h waking day period and additionally adjusting for total waking day duration.

## Results

**Fig. 1:**
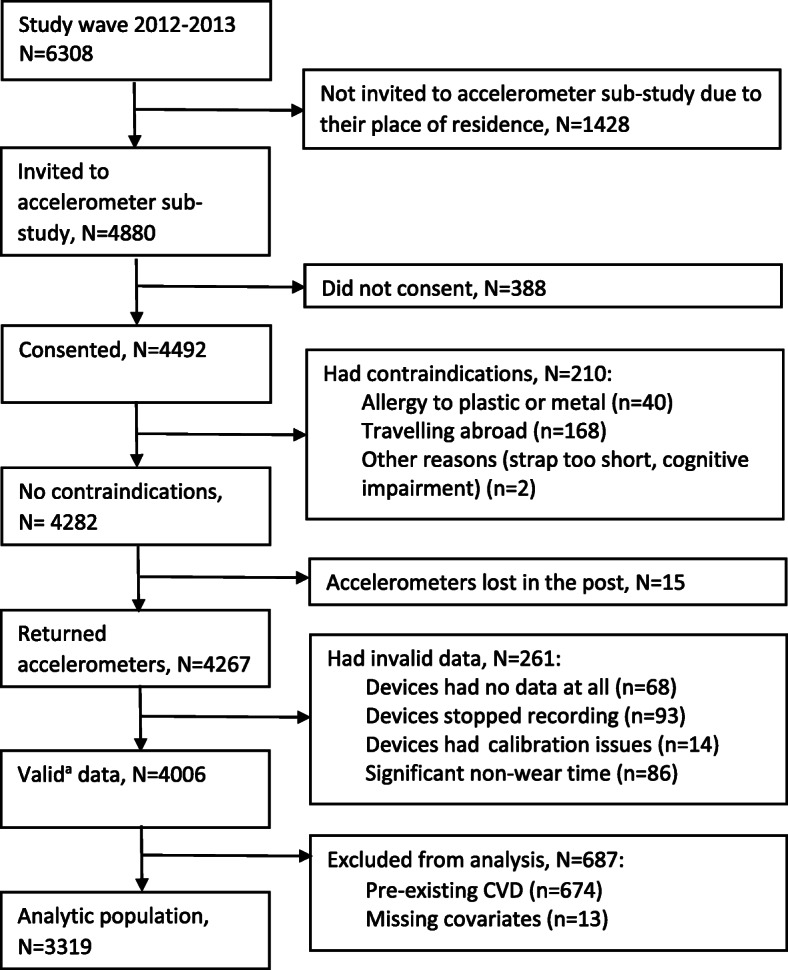
Participant flow chart. ^a^ Defined as daily wear time ≥ 2/3 of waking hours, for at least 2 weekdays and 2 week-end days

Of the 6308 participants in the 2012–2013 wave, 4880 (4680 seen at the London clinic and 200 at home) were invited to participate in the accelerometer sub-study, with 4492 agreeing and 4006 returning the device with valid data (Fig. 1). Excluding pre-existing CVD (*n* = 674; CHD (90.6%), stroke (6.4%) and heart failure (3%)) or those with missing data for covariates (*n* = 13) led to an analytical sample of 3319 participants. Compared with participants not included (*n* = 2989) in the analyses, those included (*n* = 3319) were on average younger (included vs excluded participants: 68.9 vs 70.9 years, *p* < 0.001), more likely to be men (73.3% vs 67.7%, p < 0.001) and from higher occupational position (55.4 vs 49.7%, p < 0.001). In total, 97.6% of the analytic sample had valid data for 7 days, 1.4% for 6 days, 0.6% for 5 days, and 0.5% for 4 days. Among the 3319 study participants, 299 incident CVD cases (CHD (62.9%), stroke (17.7%) and heart failure (19.4%)) were recorded over a mean follow-up of 6.2 years (standard deviation (SD) = 1.3). Participants with incident CVD were more likely to be older, men, non-white, less educated, have a worse cardiometabolic profile, and spend more time in SB and less time in LIPA and MVPA compared with participants who did not develop CVD during follow-up (Table 1).

**Table 1:** Characteristics of the study population at baseline (2012–2013) by incident CVD

	Incident CVD (***N*** = 3319)		
Characteristics	No	Yes	***P*** value
N (row %)	3020 (91.0)	299 (9.0)	
Age (years), M (SD)	68.6 (5.5)	71.5 (5.9)	< 0.001*
Women	830 (27.5)	55 (18.4)	0.001*
Non-white	173 (5.7)	33 (11.0)	< 0.001*
Married/cohabitating	2262 (74.9)	226 (75.6)	0.79
Higher education	995 (33.0)	76 (25.4)	0.01*
Low occupational position	1494 (49.5)	155 (51.8)	0.43
Recent-ex/current smokers	151 (5.0)	23 (7.7)	0.05
1–14 units of alcohol per week	1712 (56.7)	166 (55.5)	0.92
Daily intake of fruits & vegetable	2423 (80.2)	227 (75.9)	0.08
BMI (kg/m^2^), M (SD)	26.3 (4.2)	27.1 (4.4)	0.003*
Hypertension^a^	1347 (44.6)	183 (61.2)	< 0.001*
Hyperlipidaemia^b^	1365 (45.2)	150 (50.2)	0.10
Diabetes	311 (10.3)	58 (19.4)	< 0.001*
Multimorbidity index (N chronic conditions)^c^			0.67
0	2153 (71.3)	207 (69.3)	
1	742 (24.6)	77 (25.8)	
≥ 2	125 (4.1)	15 (5.0)	
Time in SB^d^ (minutes/day), M (SD)	692.1 (88.4)	718.0 (96.8)	< 0.001*
Time in LIPA^d^ (minutes/day), M (SD)	209.5 (65.1)	195.2 (73.2)	< 0.001*
Time in MVPA^d^ (minutes/day), M (SD)	58.4 (37.6)	46.8 (36.4)	< 0.001*

Notes: Values are N (column %) unless otherwise indicated

^a^Systolic/diastolic blood pressure ≥ 140/90 mmHg or use of antihypertensive drugs

^b^Low-density lipoprotein ≥4.1 mmol/l or use of lipid lowering drugs

^c^Cancer, arthritis, chronic obstructive pulmonary disease, depression, Parkinson disease, and dementia

^d^Normalized to a 16-h (960 min) waking day

* indicates statistically significance at *p* < 0.05

Abbreviations: *BMI* body mass index; *CVD* cardiovascular disease; *LIPA* light intensity physical activity; *M* mean; *MVPA* moderate-to-vigorous physical activity; *SD* standard deviation; *SB* sedentary behaviour

**Fig. 2:**
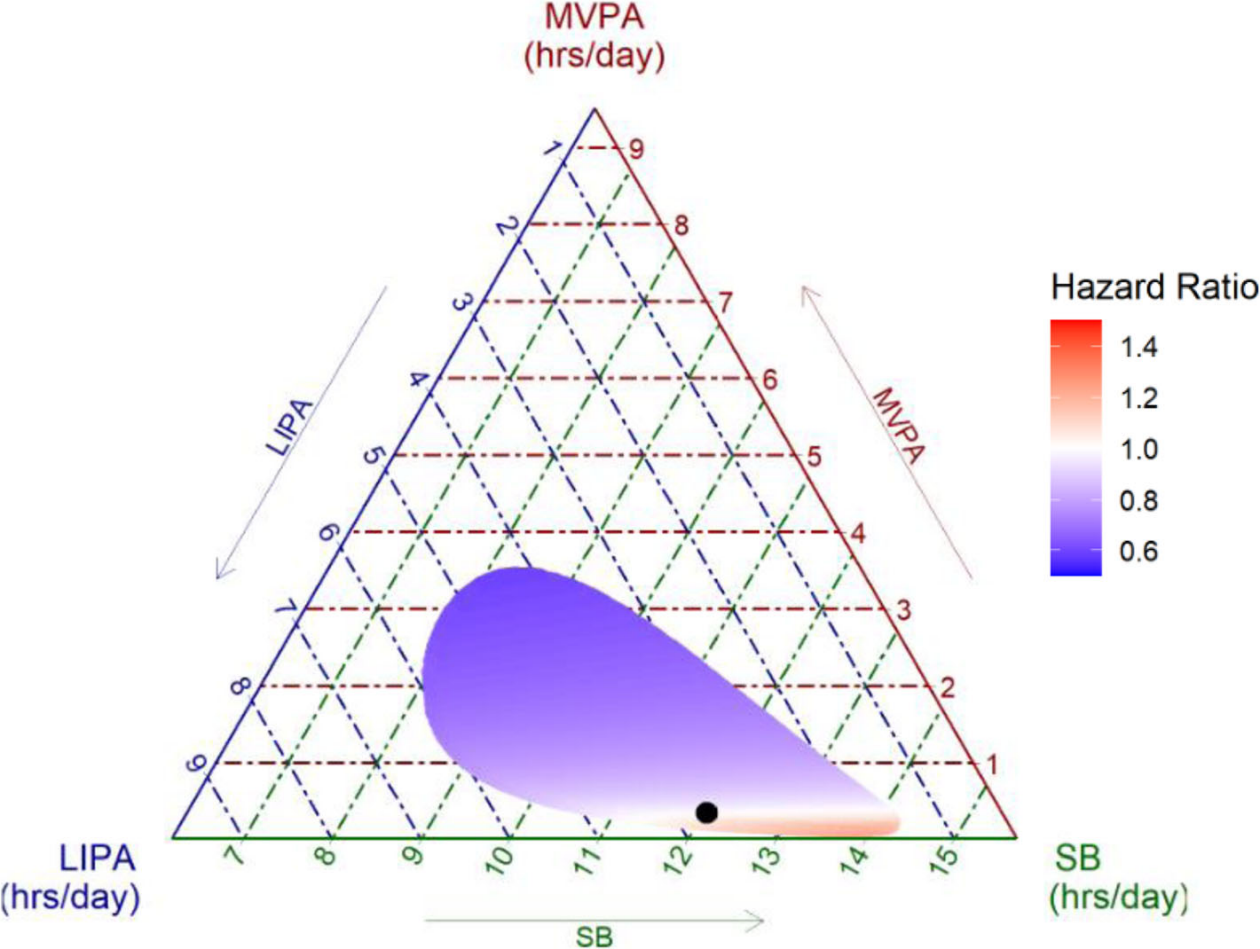
Heatmap ternary plot of the association of SB, LIPA and MVPA with CVD compared to reference movement behaviour composition indicated by black circle (SB = 12 h 2 min, LIPA = 3 h 37 min, MVPA = 21 min per day)*.* Notes: Heatmap shows hazard ratio of CVD for different movement behaviour compositions compared to the refence composition (black circle). Analyses adjusted for age, sex, ethnicity, marital status, education, occupational position, alcohol consumption, smoking status, diet, body mass index, diabetes, hypertension, hyperlipidaemia, and multimorbidity index. Range of duration of SB, LIPA and MVPA in the plot reflects observed data in the study sample. Abbreviations: *CVD* cardiovascular disease; *HR* hazard ratio; *LIPA* light intensity physical activity; *MVPA* moderate-to-vigorous physical activity; *SB* sedentary behaviour

There was no evidence of effect modification by age (*P* for interaction = 0.83), sex (*P* = 0.46), ethnicity (*P* = 0.40), and BMI (*P* = 0.14), thus analyses were conducted in the full study sample. Table 2 presents the results from compositional Cox regression on the association between daily composition of movement behaviours (SB, LIPA, MVPA) and incident CVD. The proportional hazard assumption was met. More time spent in SB relative to time spent in physical activity (LIPA and MVPA) was associated with increased risk of incident CVD (HR ${z}_1^1$ = 1.34, 95% CI: 1.01–1.79) in a model adjusted for sociodemographic and lifestyle variables. After additional adjustment for cardiometabolic risk factors and multimorbidity index, the association was no longer significant (HR 1.24, 95% CI: 0.92–1.67). Increase in MVPA relative to time spent in SB and LIPA was associated with reduced CVD risk (HR ${z}_1^3$ = 0.73, 95% CI: 0.60–0.89) when adjusted for sociodemographic and lifestyle factors, with a slight attenuation after adjusting for all covariates (HR 0.79, 95% CI: 0.64–0.97). Time spent in LIPA relative to other movement behaviours (${z}_1^2$) was not associated with CVD risk. The heatmap ternary plot (Fig. 2) illustrates the dominance of MVPA relative to other movement behaviours in reducing CVD risk.

**Table 2:** Relative importance of SB, LIPA & MVPA for incident CVD (N = 3319)

Ilr-coordinate^**a**^	Model 1^**b**^		Model 2^**c**^	
	HR (95% CI)	***P*** value	HR (95% CI)	***P*** value
**Rotation 1: relative importance of SB**				
${z}_1^1$(SB increase relative to LIPA and MVPA)	1.34 (1.01–1.79)	0.04*	1.24 (0.92–1.67)	0.16
${z}_2^1$(LIPA increase relative to MVPA)	1.21 (0.88–1.67)	0.25	1.16 (0.84–1.62)	0.36
**Rotation 2: relative importance of LIPA**				
${z}_1^2$(LIPA increase relative to SB and MVPA)	1.02 (0.68–1.51)	0.93	1.02 (0.68–1.54)	0.91
${z}_2^2$(SB increase relative to MVPA)	1.42 (1.20–1.68)	< 0.0001*	1.30 (1.09–1.56)	0.004*
**Rotation 3: relative importance of MVPA**				
${z}_1^3$(MVPA increase relative to SB and LIPA)	0.73 (0.60–0.89)	0.002*	0.79 (0.64–0.97)	0.02*
${z}_2^3$(SB increase relative to LIPA)	1.17 (0.80–1.72)	0.41	1.12 (0.75–1.66)	0.58

^a^See Methods in Additional file 1 for details of ilr coordinates z1 and z2 for all rotations

^b^Model 1 is adjusted for age, sex, ethnicity, marital status, education, occupational position, alcohol consumption, smoking

status, and diet

^c^Model 2 is additionally adjusted for body mass index, diabetes, hypertension, hyperlipidaemia, and multimorbidity index

* indicates statistically significance at *p* < 0.05

Abbreviations: *CI* confidence interval; *CVD* cardiovascular disease; HR hazard ratio; *Ilr* isometric log-ratio; *LIPA* light intensity

physical activity; *MVPA* moderate-to-vigorous physical activity; *SB* sedentary behaviour

**Fig. 3:**
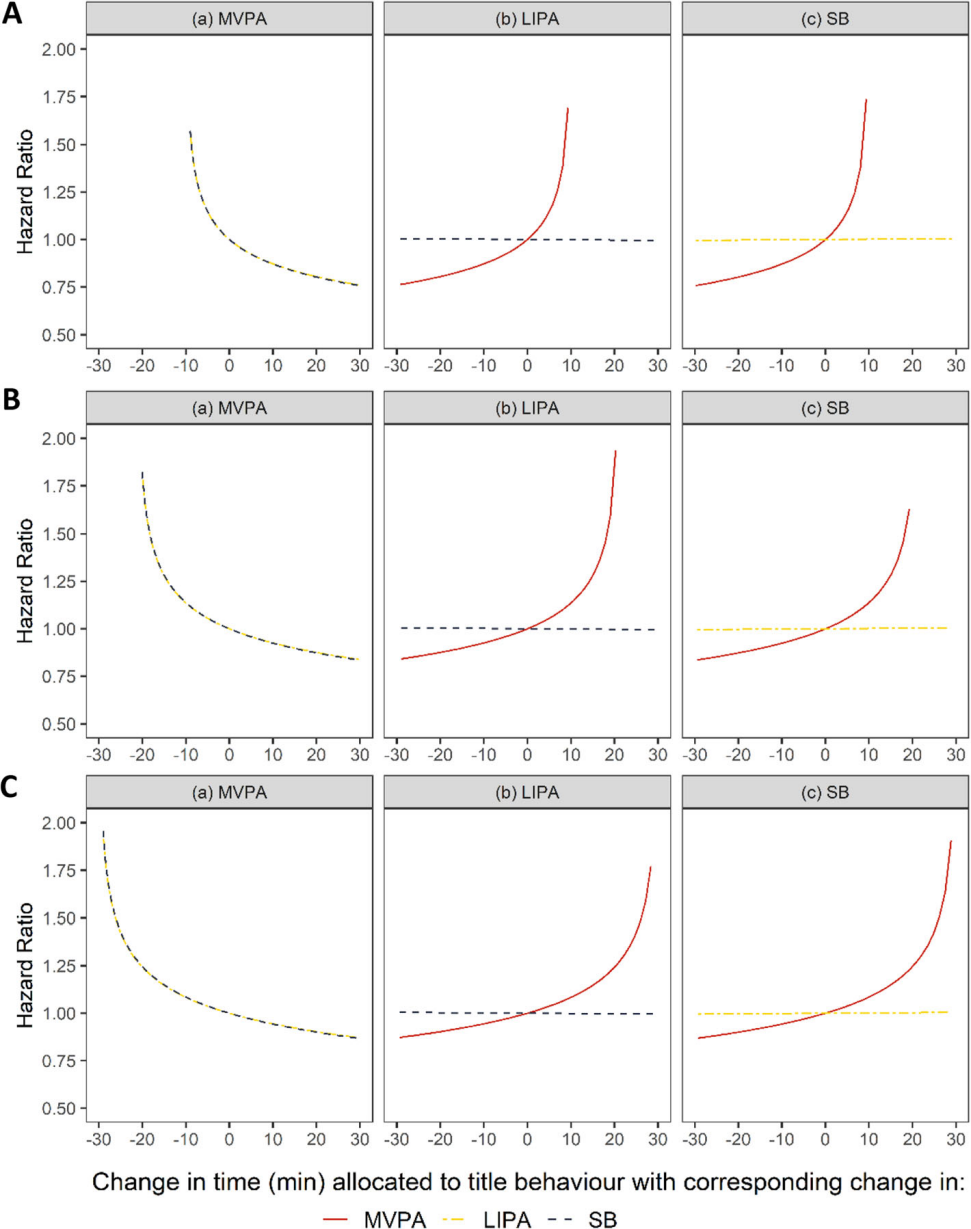
HRs for hypothetical time reallocation between movement behaviors. All analyses adjusted for sociodemographic, lifestyle, cardiometabolic risk factors and multimorbidity index. Time is displaced between title behavior (x-axis) and behavior indicated by the line, while holding the third behavior fixed with respect to reference composition. Time reallocation is modelled around reference composition values for MVPA, LIPA, SB set at (**A**) 10 min, 3h39min, 12h11min; (**B**) 21 min, 3h37min, 12h2min; (**C**) 30 min, 3h34min, 11h56min. Time reallocation were not made beyond 10 min for reference composition where MVPA is set at 10 min (**A**); and 30 min when the reference composition value is at either 21- (**B**) or 30- (**C**) minutes. Abbreviations: *HR* hazard ratio; *LIPA* light intensity physical activity; *MVPA* moderate-to-vigorous physical activity; *SB* sedentary behaviour

Figure 3 shows fully adjusted HRs for incident CVD associated with daily time reallocating from one movement behaviour to another, keeping the third fixed at the reference composition, using three reference compositions (panels A, B, C) characterised by varying MVPA duration. The estimates and their CI are detailed in Table 3 for 10-min reallocation and in Supplemental Table 1 (Additional file 2) for 20- and 30-min reallocation. Compared to a daily composition of movement behaviour made of 10 min of MVPA (reference A; SB = 12 h 11 min, LIPA = 3 h 39 min per day), a composition with 10 min less of SB and 10 min more of MVPA (SB = 12 h 1 min, LIPA = 3 h 39 min, MVPA = 20 min per day) was associated with 13% reduction in CVD risk (HR = 0.87, 95%CI: 0.78–0.98), see Table 3. Independently of time in MVPA in the reference compositions, reallocating time from MVPA to either SB or LIPA was associated with a larger risk of CVD (Fig. 3), than the reduction of risk associated with allocating the same time from either behaviour into MVPA. Compared to reference composition A, when using reference composition B and C (made of 21 and 30 min of MVPA respectively) smaller reductions in CVD risk were observed for reallocation of 10-, 20- or 30- min of MVPA to SB (Fig. 3).

**Table 3:** HRs and 95% CI for incident CVD associated with hypothetical reallocation of 10 min in daily movement behaviours: impact of MVPA duration in the reference compositions (*N* = 3319)

Reference A:^**a**^ less than recommended (MVPA = 10 min per day)			
	Add 10 min per day to:		
**Remove 10 min per day from:**	**SB**	**LIPA**	**MVPA**
**SB**	–	1.00 (0.98–1.02)	0.87 (0.78–0.98)*
**LIPA**	1.00 (0.98–1.02)	–	0.87 (0.77–0.99)*
**MVPA**	–	–	–
**Reference B:**^**a**^ **recommendation of 150 min per week (MVPA = 21 min per day)**			
	**Add 10 min per day to:**		
**Remove 10 min per day from:**	**SB**	**LIPA**	**MVPA**
**SB**	–	1.00 (0.98–1.02)	0.92 (0.87–0.99)*
**LIPA**	1.00 (0.98–1.02)	–	0.93 (0.86–1.00)*
**MVPA**	1.14 (1.02–1.27)*	1.14 (1.01–1.28)*	–
**Reference C:**^**a**^ **recommendation of 30 min per day (MVPA = 30 min per day)**			
	**Add 10 min per day to:**		
**Remove 10 min per day from:**	**SB**	**LIPA**	**MVPA**
**SB**	–	1.00 (0.98–1.02)	0.94 (0.90–0.99)*
**LIPA**	1.00 (0.98–1.02)	–	0.94 (0.89–1.00)
**MVPA**	1.09 (1.01–1.16)*	1.08 (1.00–1.17)*	–

Notes: Data represents HR (95% CI). * indicates statistically significance at *p* < 0.05. All analyses adjusted for age, sex, ethnicity, marital status, education, occupational position, alcohol consumption, smoking, status, diet, body mass index, diabetes, hypertension, hyperlipidaemia, and multimorbidity index

^**a**^ Reference compositions represent individuals undertaking 10-, 21-, or 30- min in MVPA per day and time in SB and LIPA are set proportional to population mean at 77 and 23%, respectively, of the remaining waking time as observed in the data. This corresponds to SB set at 12 h 11 min, 12 h 2 min, and 11 h 56 min, and LIPA at 3 h 39 min, 3 h 37 min, and 3 h 34 min, respectively for references A, B, and C

- Data not observed as MVPA cannot be decreased by 10 min when the reference composition value of MVPA is at 10 min

Abbreviations: *CI* confidence interval; *CVD* cardiovascular disease; *HR* hazard ratio; *LIPA* light intensity physical activity; *MVPA* moderate-to-vigorous physical activity; *SB* sedentary behaviour

Table 4 and Supplemental Table 2 (Additional file 2) show the association of daily composition of movement behaviours with incident CVD compared to two reference compositions defined by varying SB and LIPA durations for a given 10 min of MVPA. Compared to a daily composition with 9 h of SB, 6 h 50 min of LIPA and 10 min of MVPA duration (reference 1), compositions with 10 (20- or 30-) minute decrease in either SB or LIPA and equal increase in MVPA were associated with lower risk of CVD. In comparison, when using reference 2 composed with 14 h of SB, 1 h 50 min of LIPA and 10 min of MVPA duration, same risk reductions were observed for movement behaviour compositions with greater MVPA coupled with equal less time in SB, but associations were not significant when coupled with less LIPA. Similar associations were found when the reference composition had 21 min instead of 10 min of MVPA duration (Supplemental Fig. 1 in Additional file 2).

**Table 4:** HRs and 95% CI for incident CVD associated with hypothetical reallocation of 10 min in daily movement behaviours: impact of SB and LIPA duration in the reference composition for a given MVPA duration of 10 min per day (N = 3319)

**Reference** 1**:**^**a**^ **SB (9 h per day) & LIPA** (**6 h 50 min per day)**			
	**Add 10 min per day to:**		
**Remove 10 min per day from:**	**SB**	**LIPA**	**MVPA**
**SB**	–	1.00 (0.99–1.01)	0.87 (0.78–0.98)*
**LIPA**	1.00 (0.99–1.02)	–	0.87 (0.77–0.99)*
**MVPA**	–	–	–
**Reference** 2**:**^**a**^ **SB (14 h per day) & LIPA** (**1 h 50 min per day)**			
	**Add 10 min per day to:**		
**Remove 10 min per day from:**	**SB**	**LIPA**	**MVPA**
**SB**	–	1.00 (0.97–1.03)	0.87 (0.78–0.98)*
**LIPA**	1.00 (0.97–1.03)	–	0.87 (0.76–1.00)
**MVPA**	–	–	–

Notes: Data represents HR (95% CI). * indicates statistically significance at p < 0.05. All analyses adjusted for age, sex, ethnicity, marital status, education, occupational position, alcohol consumption, smoking, status, diet, body mass index, diabetes, hypertension, hyperlipidaemia, and multimorbidity index

^**a**^ Reference compositions represent individuals with SB set at 9 h and 14 h corresponding to the 5th and 95th percentile, respectively as observed in the data. Time in LIPA is set at the remaining waking time after considering time in SB and MVPA

- Data not observed as MVPA cannot be decreased by 10 min when the reference composition value of MVPA is at 10 min

Abbreviations: *CI* confidence interval; *CVD* cardiovascular disease; *HR* hazard ratio; *LIPA* light intensity physical activity; *MVPA* moderate-to-vigorous physical activity; *SB* sedentary behaviour

### Sensitivity analyses

Excluding 88 CVD events within the first 2 years of follow-up (Supplemental Table 3 in Additional file 2) attenuated the association with MVPA relative to other movement behaviours (*P* = 0.06), although the magnitude of risk reduction remains similar (HR ${z}_1^3$ = 0.79, 95% CI: 0.62–1.01). Analyses examining the association with non-fatal CVD events (Supplemental Table 4 in Additional file 2), those using an alternative cut-off to define movement behaviours (Supplemental Table 5 in Additional file 2) and those using non-normalized movement behaviours (Supplemental Table 6 in Additional file 2) yielded results similar to the main analysis (Table 2).

## Discussion

In this longitudinal study of 3319 older adults, greater MVPA duration was associated with lower risk of CVD over a mean follow-up of 6 years irrespective of duration of SB and LIPA, independent of sociodemographic, lifestyle and cardiometabolic risk factors. There was no evidence of lower CVD risk when LIPA was increased at the expense of SB. We found that decrease in MVPA duration below the current recommendations accompanied by either an increase in SB or LIPA had an adverse effect on CVD risk; the effect estimate was greater than the beneficial effect seen with increase in MVPA above current recommendations.

The present findings are consistent with previous prospective studies using self-reported [3] and objectively measured [11, 16–18] activity showing a curvilinear dose-response association between MVPA and CVD, with greatest benefit seen up to recommended MVPA duration. A meta-analysis of 33 prospective studies with mean follow-up of 12.8 years using self-reported physical activity found greatest CVD risk reduction moving from no physical activity to engaging in physical activity levels equivalent to the recommended 150 min of MVPA per week (11.25 metabolic equivalent of task (MET) h/week), with fewer benefits beyond this level [3]. One study based on 5585 middle to older aged participants followed for 5.7 years also reported a non-linear association between objectively-assessed MVPA and risk of incident CVD with the steepest decrease in risk observed among those undertaking 10 to 20 min/day of MVPA compared to none and a plateauing of the association at longer MVPA durations [16].

Our study adds to the current knowledge on the association between MVPA and CVD risk by highlighting the asymmetrical response to an increment or decrement in MVPA duration. Reduction in MVPA was found to have larger detrimental effect on CVD risk than the gains obtained from an increase in MVPA duration by fall in SB or LIPA. Some studies using similar compositional approach have also reported asymmetric association in relation to mortality [41] and cardiometabolic biomarkers [21, 42–44]. This could be explained by the rapid pace of weight gain or deconditioning with reduction in MVPA against an equivalent amount of weight loss or conditioning which takes far greater exercise effort [21].

Findings on SB using self-reported data [7, 9, 45] show greater sedentariness to be associated with increase in CVD risk, although results using objective measurements are mixed [16–20]. A meta-analysis of 9 prospective cohort studies on 720,425 participants (mean age, 54.4 years) with median follow-up of 11 years found a nonlinear association between self-reported SB and CVD risk after adjustment for physical activity, with increased risk observed only at SB duration > 10 h/day [9]. Findings from the Objective Physical Activity and Cardiovascular Health (OPACH) study on 5638 older women with mean follow-up of 4.9 years found a linear dose-response association between SB and CVD risk, after adjustment for MVPA [19]. However, three other studies did not find an association between objectively-assessed SB and incident CVD, two of these adjusted [16, 17] while another [18] did not for MVPA. Our results indicate that longer duration in SB increases risk of CVD when this is accompanied by reduction in MVPA. The magnitude of association was dependent on duration of MVPA rather than on SB unlike in a study using self-reported data [46] where replacing sitting with MVPA showed pronounced benefits for CVD mortality among those with > 6 h/day of sitting. Reasons for inconsistencies in findings across studies may relate to the nature of the measures (subjective and objective), difference in target population, and adjustment for other movement behaviours. Our study takes into account the relative co-dependency of movement behaviours within a day, unlike other studies where SB was controlled either for total wear time [18] or MVPA [19].

The association between objectively-assessed LIPA and incident CVD has been examined by a few studies but the results are inconsistent [11, 16–18, 20, 47–49]. In the OPACH study based on 5750 older women followed for 3.5 years, more LIPA was associated with decreased CVD risk independent of MVPA, although the association was attenuated upon adjustment for cardiovascular risk factors [11]. Two studies used data from the National Health and Nutrition Examination Survey (NHANES): one found increase in LIPA accompanied by an equivalent decrease in SB was associated with lower risk of CVD mortality [48], while the other did not find an association between LIPA and CVD mortality in analysis adjusted for SB and MVPA [49]. In another study on 5585 adults aged 40 to 79 years, the association between LIPA and incident CVD was attenuated after adjustment for MVPA, except higher risk of CVD when LIPA was < 3 h/day [16]. Some prospective studies found no evidence for LIPA lowering CVD risk [17, 18] as in our study. Our data show that when longer LIPA duration is coupled with shorter SB duration, increase in MVPA is beneficial either by reducing SB or LIPA. However, when LIPA duration is short and SB duration high, benefits for CVD risk are evident when MVPA is increased at the expense of SB rather than LIPA. This observation highlights the importance of considering the composition of movement behaviours rather than individual movement behaviours in isolation.

This paper adopts a behavioural approach wherein time in SB, LIPA and MVPA are at an individual’s discretion leading us to conduct analyses based on waking period. Progression to older ages tends to be accompanied with increased sleep alteration, likely to be influenced by underlying neurobiological processes [50] rather than individual choice. How circadian rhythm, a comprehensive marker of both sleep and physical activity features, is associated with risk of CVD requires further research.

The present study has several strengths including the use of objectively-assessed movement behaviours, a longitudinal design, the inclusion of men and women compared with previous prominent studies based only on one sex [17, 19], and the focus on older adults where the risk of CVD is high [51]. The innovation lies in use of methods that consider the finite time constraint and the daily composition of movement behaviours, an approach not used in previous studies on CVD risk. Our analyses were adjusted for a wide range of lifestyle and cardiovascular risk factors. A further advantage is ascertainment of cardiovascular risk factors and CVD events undertaken using multiple objective sources, including clinical examinations.

Our study has some limitations. A wrist accelerometer does not provide information on posture, not allowing differentiation between standing and sitting positions [52]. This could lead to some misclassification between SB and less intense LIPA, and discrepancies with estimates of sitting time reported by studies using thigh-worn accelerometers [52] but findings from wrist accelerometers are able to classify movement behaviours based on metabolic intensity with accuracy [40]. The Whitehall II study is an occupational cohort where study participants are healthier than the general population, although the association between cardiovascular risk factors and CVD risk has been shown to be similar to that in the general population [53]. The limited number of fatal CVD events (*n* = 10) did not allow us to analysis those cases separately. Participants were included in the analysis if they had at least 2 weekdays and 2 weekend days of accelerometer recording (97.6% of the sample has 7 days of data), which might not be reflective of the long-term physical activity pattern over the follow-up period. Although, studies suggest 4 to 6 days of recording inclusive of weekend days as optimal to reliably capture weekly habitual physical activity [54, 55]. Furthermore, good test-retest reliability was for all movement behaviours. Finally, a gold standard cut-off to identify movement behaviours in older adults does not yet exist, this may lead to variability between studies. Sensitivity analysis in our study using a different cut-off did not affect findings.

This study highlights the importance of MVPA for CVD prevention among older adults, independent of duration of SB and LIPA. Among individuals who are highly sedentary, it might be better that increase in MVPA come with reduction in SB rather than LIPA. It is also important to identify those who reduce their MVPA and encourage them to at least continue MVPA at their current durations. Overall our findings lend support to the current public health guidelines of 150 min per week of MVPA [23].

## Supplementary Information

**Additional file 1.**

**Additional file 2.**

**Additional file 3.**

## Data Availability

Whitehall II data cannot be shared publicly because of constraints dictated by the study’s ethics approval and IRB restrictions. The Whitehall II data are available for sharing within the scientific community. Researchers can apply for data access at https://www.ucl.ac.uk/epidemiology-health-care/research/epidemiology-and-public-health/research/whitehall-ii/data-sharing.

## Abbreviations

BMI: Body mass index
CHD: Coronary heart disease
CVD: Cardiovascular disease
HR: Hazard ratio
HES: Hospital episode statistics
ILR: Isometric log ratio
LDL: Low-density lipoproteins
LIPA: Light intensity physical activity
MVPA: Moderate-to-vigorous physical activity
SB: Sedentary behaviour
