# Supplementary material for: Association of daily composition of physical activity and sedentary behaviour with incidence of cardiovascular disease in older adults
Source: Int J Behav Nutr Phys Act. 2021 Jul 12;18:83. doi: 10.1186/s12966-021-01157-0 (PMC8273960; doi:10.1186/s12966-021-01157-0)
Supplement: Supplementary file 1 — Additional file 1. [file 12966_2021_1157_MOESM1_ESM.docx]

**Association of daily composition of physical activity and sedentary behaviour with incidence of cardiovascular disease in older adults**

Manasa S. Yerramalla, Duncan E. McGregor, Vincent T. V. Hees, Aurore Fayosse, Aline Dugravot, Adam G. Tabak, Mathilde Chen, Sebastien F. M. Chastin, Séverine Sabia

**Additional file 1**

**Methods**

Rotation 1 corresponds to relative importance of SB and is calculated as:

| $z^{1}=\left( \begin{aligned} z_{1}^{1} =\sqrt{\frac{2}{3}}\ln\frac{SB}{\left( LIPA.MVPA \right)^{1/2}}, \\ z_{2}^{1} =\sqrt{\frac{1}{2}}ln\frac{LIPA}{MVPA} \end{aligned} \right)$ |  |
| --- | --- |

Rotation 2 corresponds to relative importance of LIPA and is calculated as:

| $z^{2} =\left( \begin{aligned} z_{1}^{2} =\sqrt{\frac{2}{3}}\ln\frac{LIPA}{\left( SB.MVPA \right)^{1/2}}, \\ z_{2}^{2} =\sqrt{\frac{1}{2}}ln\frac{SB}{MVPA} \end{aligned} \right)$ |  |
| --- | --- |

Rotation 3 corresponds to relative importance of MVPA and is calculated as:

| $z^{3} =\left( \begin{aligned} z_{1}^{3} =\sqrt{\frac{2}{3}}\ln\frac{MVPA}{\left( SB.LIPA \right)^{1/2}}, \\ z_{2}^{3} =\sqrt{\frac{1}{2}}ln\frac{SB}{LIPA} \end{aligned} \right)$ |  |
| --- | --- |
