# Supplementary material for: Association of daily composition of physical activity and sedentary behaviour with incidence of cardiovascular disease in older adults
Source: Int J Behav Nutr Phys Act. 2021 Jul 12;18:83. doi: 10.1186/s12966-021-01157-0 (PMC8273960; doi:10.1186/s12966-021-01157-0)
Supplement: Supplementary file 2 — Additional file 2. [file 12966_2021_1157_MOESM2_ESM.docx]

**Association of daily composition of physical activity and sedentary behaviour with incidence of cardiovascular disease in older adults**

Manasa S. Yerramalla, Duncan E. McGregor, Vincent T. V. Hees, Aurore Fayosse, Aline Dugravot, Adam G. Tabak, Mathilde Chen, Sebastien F. M. Chastin, Séverine Sabia

**Additional file 2**

**Supplemental Figure 1** HRs for hypothetical time reallocation between movement behaviors. All analyses adjusted for sociodemographic, lifestyle, cardiometabolic risk factors and multimorbidity index. Time is displaced between title behavior (x-axis) and behavior indicated by the line, while holding the third behavior fixed with respect to reference composition. Time reallocation is modelled around reference composition values for MVPA, LIPA, SB set at **(1)** 21min, 6h39min, 9h; **(2)** 21min, 1h39min, 14h. Time reallocation by removing 30 minutes of MVPA was not done as the reference was set at 21 minutes.

Abbreviations: *HR* hazard ratio; *LIPA* light intensity physical activity; *MVPA* moderate-to-vigorous physical activity; *SB* sedentary behaviour


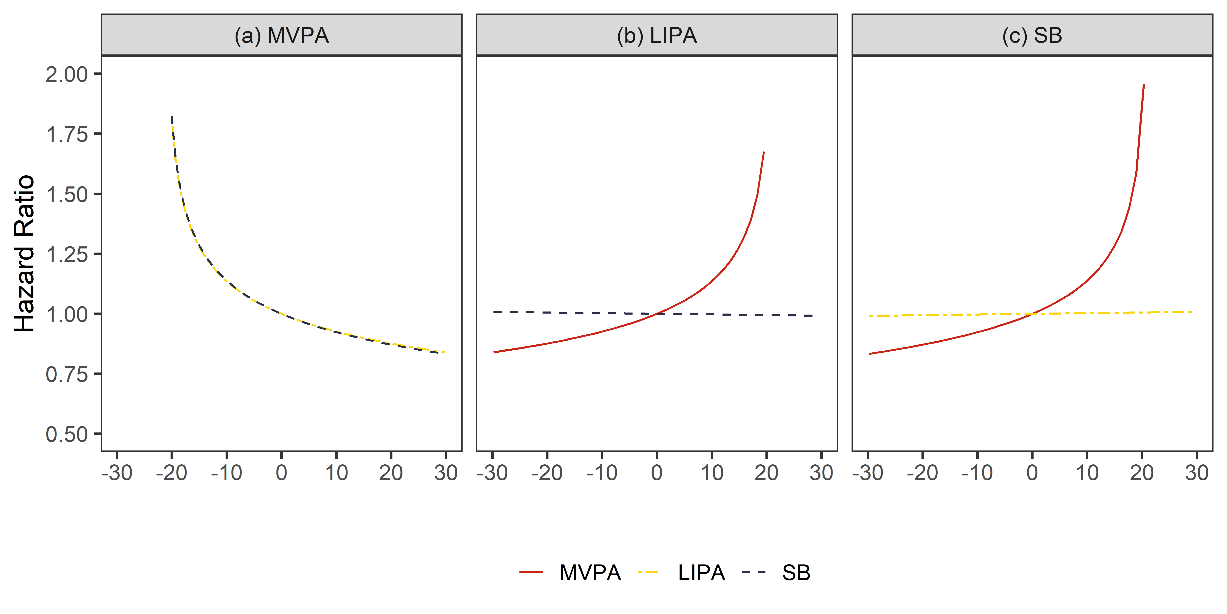


**1**

**2**


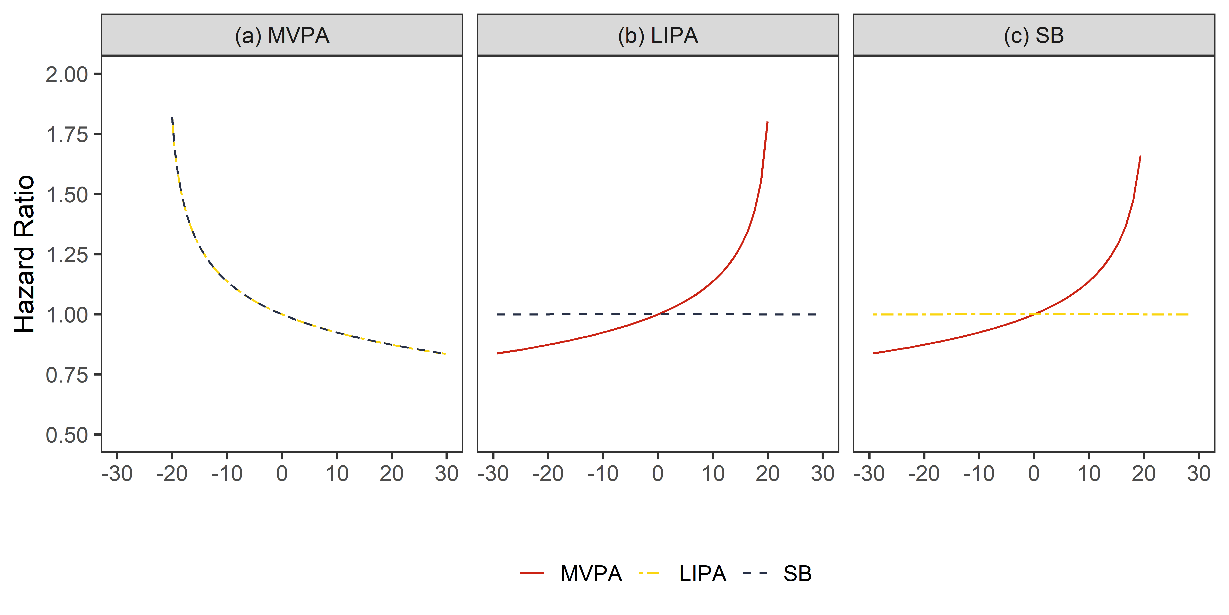

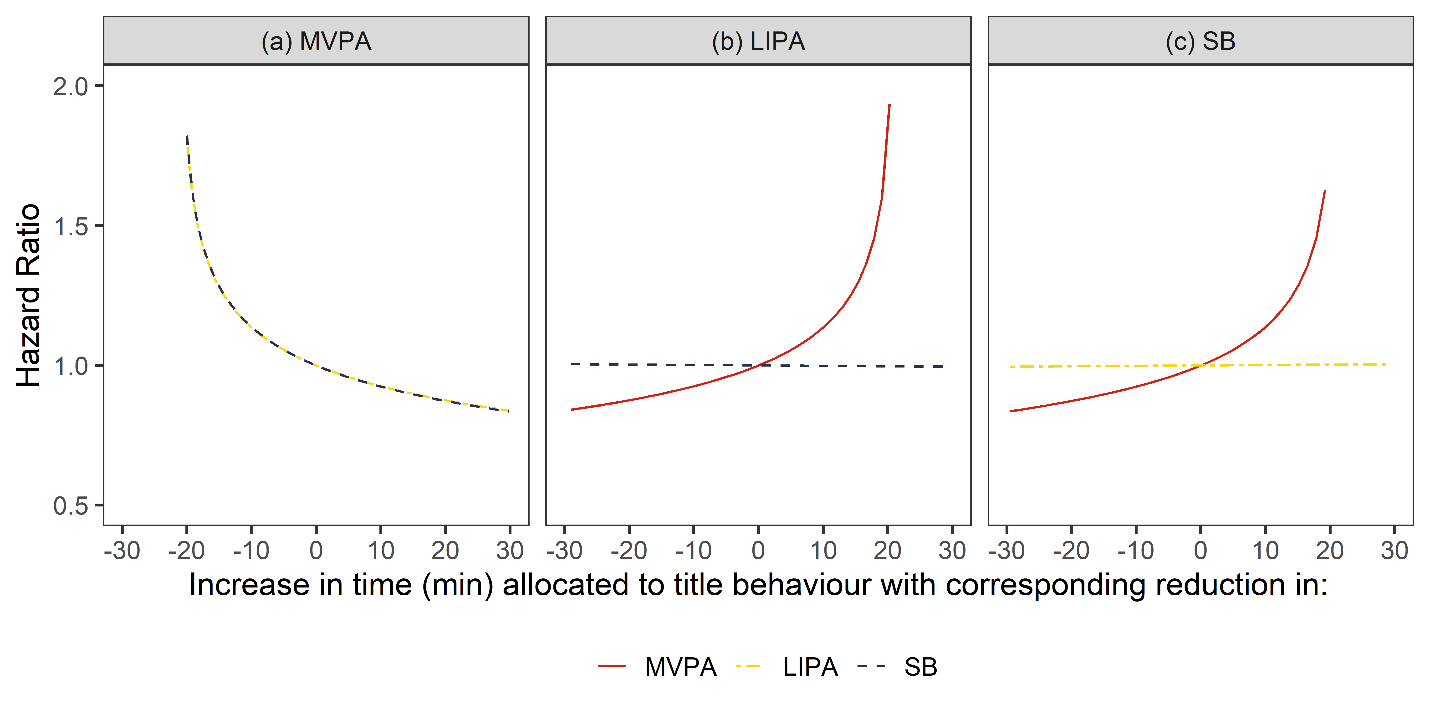

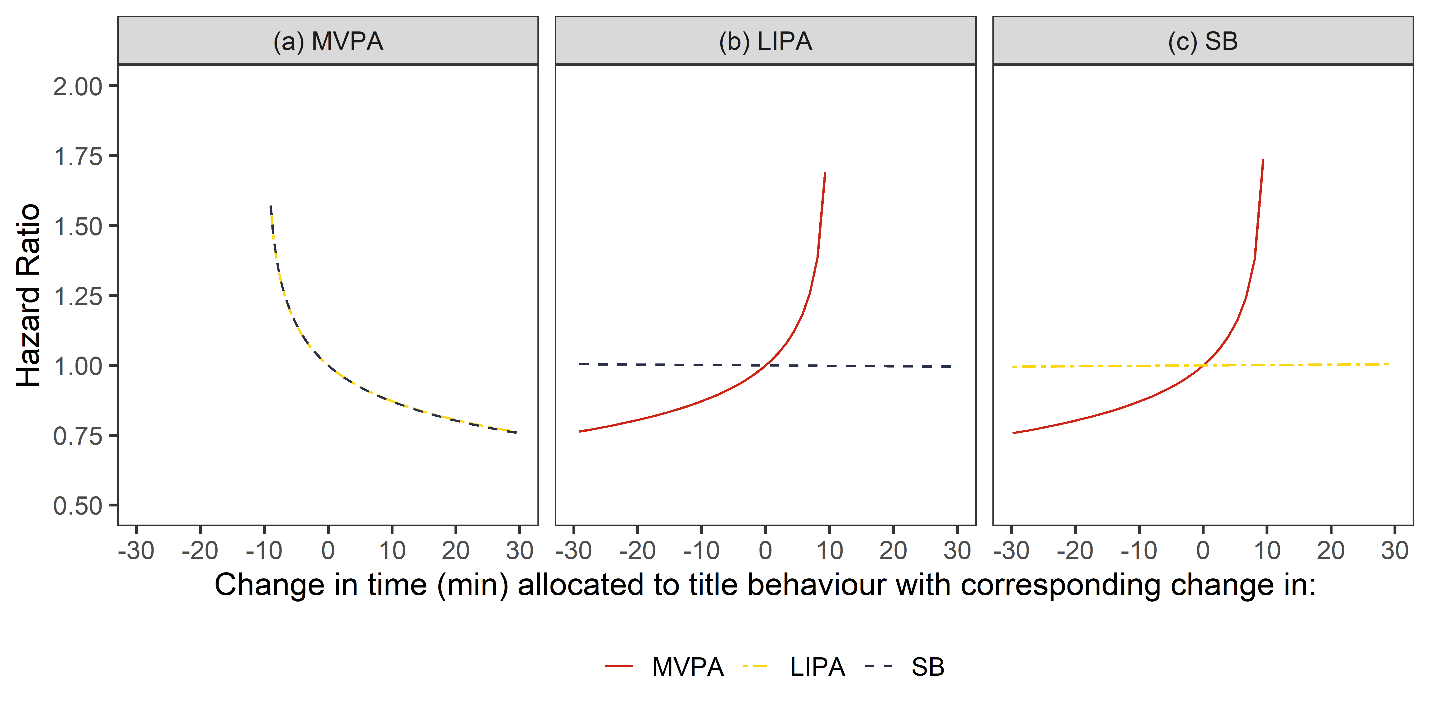


**Supplemental Table 1** HRs and 95% CI for incident CVD associated with hypothetical reallocation of 20 and 30 minutes in daily movement behaviours: impact of MVPA duration in the reference compositions (N=3319)

| **Reference A:^a^ less than recommended (MVPA = 10 min per day)** | | | | | | | | |
| --- | --- | --- | --- | --- | --- | --- | --- | --- |
|  | **Add 20 min per day to:** | | |  |  | **Add 30 min per day to:** | | |
| **Remove 20 min per day from:** | **SB** | **LIPA** | **MVPA** |  | **Remove 30 min per day from:** | **SB** | **LIPA** | **MVPA** |
| **SB** | - | 1.00 (0.96-1.03) | 0.80 (0.67-0.96)* |  | **SB** | - | 1.00 (0.95-1.05) | 0.76 (0.60-0.95)* |
| **LIPA** | 1.00 (0.97-1.04) | - | 0.81 (0.65-0.99)* |  | **LIPA** | 1.00 (0.95-1.06) | - | 0.76 (0.58-0.99)* |
| **MVPA** | -- | -- | - |  | **MVPA** | -- | -- | - |
| **Reference B:^a^ recommendation of 150 min per week (MVPA = 21 min per day)** | | | | | | | | |
|  | **Add 20 min per day to:** | | |  |  | **Add 30 min per day to:** | | |
| **Remove 20 min per day from:** | **SB** | **LIPA** | **MVPA** |  | **Remove 30 min per day from:** | **SB** | **LIPA** | **MVPA** |
| **SB** | - | 1.00 (0.96-1.03) | 0.87 (0.78-0.97)* |  | **SB** | - | 1.00 (0.94-1.05) | 0.83 (0.72-0.97)* |
| **LIPA** | 1.00 (0.97-1.04) | - | 0.88 (0.76-1.00) |  | **LIPA** | 1.00 (0.95-1.06) | - | 0.84 (0.70-1.01) |
| **MVPA** | 1.82 (1.10-3.03)* | 1.82 (1.07-3.09)* | - |  | **MVPA** | -- | -- | - |
| **Reference C:^a^ recommendation of 30 min per day (MVPA = 30 min per day)** | | | | | | | | |
|  | **Add 20 min per day to:** | | |  |  | **Add 30 min per day to:** | | |
| **Remove 20 min per day from:** | **SB** | **LIPA** | **MVPA** |  | **Remove 30 min per day from:** | **SB** | **LIPA** | **MVPA** |
| **SB** | - | 1.00 (0.96-1.03) | 0.90 (0.83-0.98)* |  | **SB** | - | 1.00 (0.94-1.05) | 0.87 (0.77-0.97)* |
| **LIPA** | 1.00 (0.97-1.04) | - | 0.90 (0.81-1.01) |  | **LIPA** | 1.00 (0.95-1.07) | - | 0.87 (0.75-1.02) |
| **MVPA** | 1.25 (1.04-1.49)* | 1.24 (1.01-1.53)* | - |  | **MVPA** | -- | -- | - |

Notes: Data represents HR (95% CI). * indicates statistically significance at p < 0.05

All analyses adjusted for age, sex, ethnicity, marital status, education, occupational position, alcohol consumption, smoking, status, diet, body mass index, diabetes, hypertension, hyperlipidaemia, and multimorbidity index

**^a^** Reference compositions represent individuals undertaking 10-, 21-, or 30- minutes in MVPA per day and time in SB and LIPA are set proportional to population mean at 77% and 23%, respectively, of the remaining waking time as observed in the data. This corresponds to SB set at 12h 11min, 12h 2min, and 11h 56min, and LIPA at 3h 39min, 3h 37min, and 3h 34min, respectively for references A, B, and C

-- Data not observed as MVPA cannot be decreased by 30 minutes when the reference composition value of MVPA is at either 21- or 30- minutes

Abbreviations: *CI* confidence interval; *CVD* cardiovascular disease; *HR* hazard ratio; *LIPA* light intensity physical activity; *MVPA* moderate-to-vigorous physical activity; *SB* sedentary behaviour

**Supplemental Table 2** HRs and 95% CI for incident CVD associated with hypothetical reallocation of 20 and 30 minutes in daily movement behaviours: impact of SB and LIPA duration in the reference composition for a given MVPA duration of 10 minutes per day (N=3319)

| **Reference 1:^a^ SB (9h per day) & LIPA** (**6 h 50 min per day)** | | | | | | | | |
| --- | --- | --- | --- | --- | --- | --- | --- | --- |
|  | **Add 20 min per day to:** | | |  |  | **Add 30 min per day to:** | | |
| **Remove 20 min per day from:** | **SB** | **LIPA** | **MVPA** |  | **Remove 30 min per day from:** | **SB** | **LIPA** | **MVPA** |
| **SB** | - | 0.99 (0.97-1.02) | 0.80 (0.67-0.96)* |  | **SB** | - | 0.99 (0.96-1.03) | 0.75 (0.60-0.95)* |
| **LIPA** | 1.01 (0.98-1.03) | - | 0.81 (0.66-0.98)* |  | **LIPA** | 1.01 (0.97-1.05) | - | 0.76 (0.59-0.98)* |
| **MVPA** | -- | -- | - |  | **MVPA** | -- | -- | - |
| **Reference 2:^a^ SB (14h per day) & LIPA** (**1 h 50 min per day)** | | | | | | | | |
|  | **Add 20 min per day to:** | | |  |  | **Add 30 min per day to:** | | |
| **Remove 20 min per day from:** | **SB** | **LIPA** | **MVPA** |  | **Remove 30 min per day from:** | **SB** | **LIPA** | **MVPA** |
| **SB** | - | 1.00 (0.94-1.06) | 0.80 (0.67-0.96)* |  | **SB** | - | 1.00 (0.91-1.09) | 0.76 (0.60-0.95)* |
| **LIPA** | 1.00 (0.93-1.07) | - | 0.80 (0.63-1.02) |  | **LIPA** | 1.00 (0.89-1.12) | - | 0.76 (0.55-1.04) |
| **MVPA** | -- | -- | - |  | **MVPA** | -- | -- | - |

Notes: Data represents HR (95% CI). * indicates statistically significance at p < 0.05. All analyses adjusted for age, sex, ethnicity, marital status, education, occupational position, alcohol consumption, smoking, status, diet, body mass index, diabetes, hypertension, hyperlipidaemia, and multimorbidity index

**^a^** Reference compositions represent individuals with SB set at 9h and 14h corresponding to the 5^th^ and 95^th^ percentile, respectively as observed in the data. Time in LIPA is set at the remaining waking time after considering time in SB and MVPA

-- Data not observed as MVPA cannot be decreased by 20- or 30- minutes when the reference composition value of MVPA is at 10 minutes

Abbreviations: *CI* confidence interval; *CVD* cardiovascular disease; *HR* hazard ratio; *LIPA* light intensity physical activity; *MVPA* moderate-to-vigorous physical activity; *SB* sedentary behaviour

**Supplemental Table 3** Relative importance of SB, LIPA, & MVPA for incident CVD excluding CVD events occurring within

first 2 years of follow-up (N cases/total N=211/3231)

| **Ilr-coordinate^a^** | **Model 1^b^** | |  | **Model 2^c^** | |  |
| --- | --- | --- | --- | --- | --- | --- |
|  | **HR (95% CI)** | ***P* value** |  | **HR (95% CI)** | ***P* value** | |
| **Rotation 1: relative importance of SB** | | | | | | |
| $z_{1}^{1}$ (SB increase relative to LIPA and MVPA) | 1.28 (0.90-1.81) | 0.16 |  | 1.16 (0.81-1.67) | 0.41 | |
| $z_{2}^{1}$ (LIPA increase relative to MVPA) | 1.23 (0.83-1.80) | 0.30 |  | 1.20 (0.81-1.78) | 0.37 | |
| **Rotation 2: relative importance of LIPA** | | | | | | |
| $z_{1}^{2}$ (LIPA increase relative to SB and MVPA) | 1.05 (0.65-1.70) | 0.83 |  | 1.09 (0.67-1.77) | 0.74 | |
| $z_{2}^{2}$ (SB increase relative to MVPA) | 1.37 (1.12-1.68) | <0.01* |  | 1.25 (1.00-1.55) | 0.05* | |
| **Rotation 3: relative importance of MVPA** | | | | | | |
| $z_{1}^{3}$ (MVPA increase relative to SB and LIPA) | 0.74 (0.59-0.94) | 0.01* |  | 0.79 (0.62-1.01) | 0.06 | |
| $z_{2}^{3}$ (SB increase relative to LIPA) | 1.12 (0.70-1.78) | 0.64 |  | 1.04 (0.65-1.67) | 0.87 | |

^a^Model 1 is adjusted for age, sex, ethnicity, marital status, education, occupational position, alcohol consumption, smoking

status, and diet

^b^Model 2 is additionally adjusted for body mass index, diabetes, hypertension, hyperlipidaemia, and multimorbidity index

^c^See Methods in Additional file 1 for details of ilr coordinates z1 and z2 for all rotations

* indicates statistically significance at p<0.05

Abbreviations: *CI* confidence interval; *CVD* cardiovascular disease; HR hazard ratio; *Ilr* isometric log-ratio; *LIPA* light intensity

physical activity; *MVPA* moderate-to-vigorous physical activity; *SB* sedentary behaviour

**Supplemental Table 4** Relative importance of SB, LIPA, & MVPA for incident non-fatal CVD events (N cases/total N=289/3319)

| **Ilr-coordinate^a^** | **Model 1^b^** | |  | **Model 2^c^** | |
| --- | --- | --- | --- | --- | --- |
|  | **HR (95% CI)** | ***P* value** |  | **HR (95% CI)** | ***P* value** |
| **Rotation 1: relative importance of SB** | | | | | |
| $z_{1}^{1}$ (SB increase relative to LIPA and MVPA) | 1.31 (0.98-1.76) | 0.07 |  | 1.20 (0.89-1.63) | 0.24 |
| $z_{2}^{1}$ (LIPA increase relative to MVPA) | 1.19 (0.86-1.66) | 0.29 |  | 1.14 (0.82-1.60) | 0.44 |
| **Rotation 2: relative importance of LIPA** | | | | | |
| $z_{1}^{2}$ (LIPA increase relative to SB and MVPA) | 1.02 (0.68-1.53) | 0.93 |  | 1.02 (0.68-1.55) | 0.91 |
| $z_{2}^{2}$ (SB increase relative to MVPA) | 1.38 (1.16-1.64) | <0.001* |  | 1.25 (1.04-1.51) | 0.02* |
| **Rotation 3: relative importance of MVPA** | | | | | |
| $z_{1}^{3}$ (MVPA increase relative to SB and LIPA) | 0.75 (0.61- 0.92) | 0.01* |  | 0.81 (0.66-1.00) | 0.05 |
| $z_{2}^{3}$ (SB increase relative to LIPA) | 1.16 (0.78-1.71) | 0.47 |  | 1.10 (0.73-1.64) | 0.65 |

^a^Model 1 is adjusted for age, sex, ethnicity, marital status, education, occupational position, alcohol consumption, smoking

status, and diet

^b^Model 2 is additionally adjusted for body mass index, diabetes, hypertension, hyperlipidaemia, and multimorbidity index

^c^See Methods in Additional file 1 for details of ilr coordinates z1 and z2 for all rotations

* indicates statistically significance at p<0.05

Abbreviations: *CI* confidence interval; *CVD* cardiovascular disease; HR hazard ratio; *Ilr* isometric log-ratio; *LIPA* light intensity

physical activity; *MVPA* moderate-to-vigorous physical activity; *SB* sedentary behaviour

**Supplemental Table 5** Relative importance of SB, LIPA, & MVPA for incident CVD (N=3319) using cut-off <45 mg for SB,

45-99 mg for LIPA and ≥100 mg for MVPA (N=3319)

| **Ilr-coordinate^a^** | **Model 1^b^** | |  | **Model 2^c^** | |
| --- | --- | --- | --- | --- | --- |
|  | **HR (95% CI)** | **P value** |  | **HR (95% CI)** | **P value** |
| **Rotation 1: relative importance of SB** | | | | | |
| $z_{1}^{1}$ (SB increase relative to LIPA and MVPA) | 1.31 (1.00-1.72) | 0.05* |  | 1.21 (0.91-1.61) | 0.18 |
| $z_{2}^{1}$ (LIPA increase relative to MVPA) | 1.26 (0.91-1.74) | 0.17 |  | 1.20 (0.86-1.68) | 0.27 |
| **Rotation 2: relative importance of LIPA** | | | | | |
| $z_{1}^{2}$ (LIPA increase relative to SB and MVPA) | 1.06 (0.72-1.57) | 0.76 |  | 1.07 (0.71-1.59) | 0.75 |
| $z_{2}^{2}$ (SB increase relative to MVPA) | 1.42 (1.21-1.67) | <0.0001* |  | 1.30 (1.09-1.55) | 0.004* |
| **Rotation 3: relative importance of MVPA** | | | | | |
| $z_{1}^{3}$ (MVPA increase relative to SB and LIPA) | 0.72 (0.58-0.88) | 0.002* |  | 0.77 (0.62-0.96) | 0.02* |
| $z_{2}^{3}$ (SB increase relative to LIPA) | 1.13 (0.78-1.64) | 0.52 |  | 1.08 (0.74-1.58) | 0.70 |

^a^Model 1 is adjusted for age, sex, ethnicity, marital status, education, occupational position, alcohol consumption, smoking

status, and diet

^b^Model 2 is additionally adjusted for body mass index, diabetes, hypertension, hyperlipidaemia, and multimorbidity index

^c^See Methods in Additional file 1 for details of ilr coordinates z1 and z2 for all rotations

* indicates statistically significance at p<0.05

Abbreviations: *CI* confidence interval; *CVD* cardiovascular disease; HR hazard ratio; *Ilr* isometric log-ratio; *LIPA* light intensity

physical activity; *MVPA* moderate-to-vigorous physical activity; *SB* sedentary behaviour

**Supplemental Table 6** Relative importance of SB, LIPA, & MVPA for incident CVD without normalization to a 16-hour waking day

| **Ilr-coordinate^a^** | **Model 1^b^** | |  | **Model 2^c^** | |
| --- | --- | --- | --- | --- | --- |
|  | **HR (95% CI)** | ***P* value** |  | **HR (95% CI)** | ***P* value** |
| **Rotation 1: relative importance of SB** | | | | | |
| $z_{1}^{1}$ (SB increase relative to LIPA and MVPA) | 1.36 (1.02-1.81) | 0.04* |  | 1.25 (0.93-1.68) | 0.14 |
| $z_{2}^{1}$ (LIPA increase relative to MVPA) | 1.19 (0.87-1.64) | 0.27 |  | 1.15 (0.83-1.59) | 0.40 |
| **Rotation 2: relative importance of LIPA** | | | | | |
| $z_{1}^{2}$ (LIPA increase relative to SB and MVPA) | 1.00 (0.68-1.49) | 0.99 |  | 1.01 (0.67-1.51) | 0.97 |
| $z_{2}^{2}$ (SB increase relative to MVPA) | 1.42 (1.20-1.68) | <0.0001* |  | 1.30 (1.09-1.56) | 0.004* |
| **Rotation 3: relative importance of MVPA** | | | | | |
| $z_{1}^{2}$ (LIPA increase relative to SB and MVPA) | 0.74 (0.61-0.89) | 0.002* |  | 0.79 (0.65-0.97) | 0.03* |
| $z_{2}^{2}$ (SB increase relative to LIPA) | 1.19 (0.81-1.75) | 0.37 |  | 1.13 (0.77-1.67) | 0.53 |

^a^Model 1 is adjusted for age, sex, ethnicity, marital status, education, occupational position, alcohol consumption, smoking

status, diet and total waking day duration

^b^Model 2 is additionally adjusted for body mass index, diabetes, hypertension, hyperlipidaemia, and multimorbidity index

^c^See Methods in Additional file 1 for details of ilr coordinates z1 and z2 for all rotations

* indicates statistically significance at p<0.05

Abbreviations: *CI* confidence interval; *CVD* cardiovascular disease; HR hazard ratio; *Ilr* isometric log-ratio; *LIPA* light intensity

physical activity; *MVPA* moderate-to-vigorous physical activity; *SB* sedentary behaviour
